# Altered Gut Microbiota and Compositional Changes in *Firmicutes* and *Proteobacteria* in Mexican Undernourished and Obese Children

**DOI:** 10.3389/fmicb.2018.02494

**Published:** 2018-10-16

**Authors:** Eder Orlando Méndez-Salazar, María Guadalupe Ortiz-López, María de los Ángeles Granados-Silvestre, Berenice Palacios-González, Marta Menjivar

**Affiliations:** ^1^Unidad de Genómica de Poblaciones Aplicada a la Salud, Facultad de Química, Universidad Nacional Autónoma de México – Instituto Nacional de Medicina Genómica, Mexico City, Mexico; ^2^Laboratorio de Endocrinología Molecular, Hospital Juárez de México, Mexico City, Mexico; ^3^Facultad de Química, Universidad Nacional Autónoma de México, Mexico City, Mexico; ^4^Unidad de Vinculación Científica de la Facultad de Medicina UNAM-INMEGEN, Instituto Nacional de Medicina Genómica, Mexico City, Mexico

**Keywords:** Mexican children, microbiota, *Firmicutes*, undernourished, obesity

## Abstract

Mexico is experiencing an epidemiological and nutritional transition period, and Mexican children are often affected by the double burden of malnutrition, which includes undernutrition (13.6% of children) and obesity (15.3%). The gut microbiome is a complex and metabolically active community of organisms that influences the host phenotype. Although previous studies have shown alterations in the gut microbiota in undernourished children, the affected bacterial communities remain unknown. The present study investigated and compared the bacterial richness and diversity of the fecal microbiota in groups of undernourished (*n* = 12), obese (*n* = 12), and normalweight (control) (*n* = 12) Mexican school-age children. We used next-generation sequencing to analyze the V3–V4 region of the bacterial 16S rRNA gene, and we also investigated whether there were correlations between diet and relevant bacteria. The undernourished and obese groups showed lower bacterial richness and diversity than the normalweight group. Enterotype 1 correlated positively with dietary fat intake in the obese group and with carbohydrate intake in the undernourished group. The results showed that undernourished children had significantly higher levels of bacteria in the *Firmicutes* phylum and in the *Lachnospiraceae* family than obese children, while the *Proteobacteria* phylum was overrepresented in the obese group. The level of *Lachnospiraceae* correlated negatively with energy consumption and positively with leptin level. This is the first study to examine the gut microbial community structure in undernourished and obese Mexican children living in low-income neighborhoods. Our analysis revealed distinct taxonomic profiles for undernourished and obese children.

## Introduction

The human gut microbiome is considered a metabolic organ due to its myriad benefits (O’Hara and Shanahan, 2006), which include the degradation of indigestible dietary polysaccharides into short-chain fatty acids (SCFAs) by fermentative enzymes in the microbiome (Flint et al., 2008; den Besten et al., 2013). Furthermore, several types of bacteria in the distal intestine can synthesize vitamins and essential amino acids (Metges, 2000; Martens et al., 2002; Morowitz et al., 2011). The gut microbiota matures around 3 years after birth. At that time, it is characterized by the presence of anaerobic microorganisms and is dominated by two bacterial phyla, *Firmicutes* and *Bacteroidetes*, which account for more than 90% of the gut microbiota population (Matamoros et al., 2013; Meehan and Beiko, 2014). When the bacterial ecosystem is disrupted (dysbiosis), some of the benefits of this metabolic organ may be reduced, and the microbiome may even harm the host (Hawrelak and Myers, 2004; Larsen and Dai, 2015). Early-life exposures, including those known to impact the composition of the gut microbiome, have been associated with an increased risk of childhood obesity, which can impact the subsequent development of obesity, type 2 diabetes, hypertension, and dyslipidemia in adulthood (Munyaka et al., 2014; Wallace et al., 2016). The gut microbiome also regulates obesity by increasing the energy harvest from the diet and by regulating peripheral metabolism (Yamashiro et al., 2015). In fact, the gut microbiota of individuals who are obese seem to have less bacterial richness, an increased level of *Firmicutes*, and a reduced level of *Bacteroidetes* (Turnbaugh et al., 2006; Le Chatelier et al., 2013). Notably, elevated fat consumption stimulates a bloom of *Proteobacteria*. The abundance of these bacterial lineages has been suggested as a potential diagnostic criterion for dysbiosis (Hildebrandt et al., 2009). Children with moderate and severe undernutrition have gut microbiota that are less mature and less diverse than those of healthy controls (Subramanian et al., 2014). Consequently, dysbiosis of gut microbiota is linked to undernutrition (Ghosh et al., 2014). Notably, gnotobiotic mice who received fecal microbiota transplants from children with kwashiorkor showed marked weight loss and metabolic abnormalities, including disturbances in amino acid, carbohydrate, and intermediary metabolism. This finding supports the idea that the gut microbiome is implicated in this type of severe undernutrition (Smith et al., 2013; Kane et al., 2015). Stunting, or low height-for-age, is the most common form of undernutrition and is very prevalent in resource-limited areas of the world. Stunting is considered the main indicator of childhood undernutrition, and the etiology of stunting is poorly understood (Ahmed et al., 2014; Dinh et al., 2016). Several studies have reported associations between stunting and altered body composition and fat distribution that predispose individuals to excess adiposity and abdominal fat distribution, which is strongly associated with the development of obesity, insulin resistance, and diabetes (Lear et al., 2009; Wilson et al., 2012; Pomeroy et al., 2014; Hardikar et al., 2015).

Mexico is going through a nutritional transition that affects school-age children, who bear the double burden of malnutrition, which includes undernutrition and obesity (13.6% and 15.3%, respectively) (Shamah-Levy et al., 2017). An undernourished population often moves from undernutrition to overnutrition, resulting in weight gain and central adiposity (Barquera et al., 2007). There is a risk of obesity in populations who move from famine early in life to abundance or even to excessive nutrition in adulthood (Martorell et al., 2001; Tzioumis and Adair, 2014). The mechanisms underlying these alterations remain unknown. However, low-income families may have an obesogenic environment due to the enrichment of sugar and edible oils in inexpensive food, resulting in a diet that is energy dense but micronutrient-poor that could alter their gut microbiome (Sawaya et al., 2003; David et al., 2014; Doak et al., 2016).

Considering this context, describing the gut microbiota in nutrition-related diseases may provide insights into the roles of microbiota in the pathogenesis of undernutrition and in obesity risk over time (Gordon et al., 2012). Thus, the aim of the present study was to describe and compare the richness and diversity of the intestinal microbiota in undernourished and obese Mexican school-age children living in low-income (marginal) and vulnerable communities (Vite-Pérez and Pérez-Zamorano, 2010; Moreno-Sánchez and Espejel-Mena, 2013).

## Materials and Methods

### Subjects

The children recruited in this study were selected from a cohort of 1,000 children attending to public schools located in Chimalhuacán, State of México. Thirty-six children aged between 9 and 11 years old belonging to low-income families, were selected and assigned to the study groups. Weight and length/height were measured and nutritional status was defined by height-for-age (HAZ) score, using the 2009 WHO child growth standards as a reference (World Health Organization United Nations Children’s Fund, 2009).

Children were classified as stunted if their HAZ score was more than two standard deviations below the median of the WHO reference standard. Obesity was considered as *z*-score ≥ +2 standard deviations, according to the cut-offs determined by WHO (de Onis et al., 2007). Normal weight group was defined according to BMI *z*-score as well. Children with any of the exclusion criteria below were not eligible for entry for the present study: antibiotic therapy or hospitalization history (>24 h) anytime 6 months prior to the study, any gastrointestinal or underlying pathology, any chronic illness, any infection requiring antibiotic therapy, diarrheal disease (World Health Organization definition) during the month prior to the study and gastro-intestinal-related medication (antibiotics prescription). The parents or legal guardians were interviewed in order to obtain socioeconomic and demographic information. The study was performed according to the latest version of the Declaration of Helsinki and was approved by the Human Research Ethical Committee of Hospital Juarez de México. All parents or legal guardians and children provided written informed consent.

### Anthropometric and Biochemical Measurements

The following anthropometric measurements were obtained: weight, height, waist circumference, tricipital and subscapular skinfolds using standardized techniques applied by trained personnel. All measurements were obtained with children wearing light clothing and no shoes. Height was measured with the child standing up and barefoot, using a stadiometer with a support and an inextensible ruler. The participants stood up erect, with arms along the body and head on the Frankfurt plane.

The tricipital skinfold was measured in the posterior midpoint of the arm between the acromion and olecranon and the subscapular skinfold was measured 2 cm below the margin of the lower angle of the scapula using a caliper (Lange Skinfold Caliper, Beta Technology, Santa Cruz, CA, United States) with a limit measurement of 40 mm. Body fat percent evaluation for girls and boys was derived from triceps and subscapular skinfold using prepubescent Slaughter equation (Kavak, 2006).

Fasting blood samples were collected and kept at 2–8°C, centrifuged within the first 15 min and stored at -80°C, until their processing in the Molecular Endocrinology Laboratory of Hospital Juárez de México. Glucose, triglycerides, total cholesterol, LDL-cholesterol and HDL-cholesterol, hepatic transaminases, prealbumin and transferrin serum levels were determined by automatic chemistry analyzer (Advia 1800 Siemens, Atlanta, EEUU). Insulin serum levels were determined by electrochemiluminescence using an Elecsys 2010 equipment (Roche^®^ Diagnostics Corp., Indianapolis, IN, United States). Both leptin and adiponectin serum levels were measured by a commercial ELISA kit, following manufacturer’s instructions (Human Leptin Quantikine and Adiponectin/Acrp30, R&D Systems, respectively).

### Dietary Assessment

Dietary intake data were obtained by using unannounced 24-h dietary recalls. The assessment of energy intake, macronutrients and micronutrients were examined through NutriKcal^®^ VO software (Consinfo, S.C., Mexico) validated for Mexican population.

### DNA Extraction and Preparation of 16S rRNA Gene Amplicon Libraries

The parent of each child was instructed to collect the first bowel movement of the day. After collection, fecal samples, placed in a sterile polypropylene container and immediately transported to the laboratory facilities in ice-filled coolers. Aliquots with 200 mg were made and stored at -80°C until processing. Bacterial DNA was extracted by using QIAamp DNA stool kit (QIAGEN, Hilden, Germany) following manufacturer’s instructions. DNA concentrations were measured by using Nano Drop V3.8.1 spectrophotometer. The V3–V4 hypervariable region of bacterial 16S rRNA gene was amplified by GeneAmp PCR system 9700 (Applied Biosystems). The First PCR conditions were as follows: 5 min at 95°C, followed by 30 cycles of 1 min at 95°C, 1 min at 45°C, and 30 s at 72°C; and a final 5 min extension at 72°C. Each 50 μL PCR reaction contained 10 ng DNA, 1 μL of each universal primers (319F/806R), 13 μL DNAse free water and 25 μL Platinum Super Mix (Invitrogen, Carlsbad, CA, United States). PCR products were purified by using magnetic AMPure XP Beads (Beckman Coulter, Danvers, MA, United States), and then quantified using the Qubit system (Invitrogen, United States) according to the manufacturer’s specifications. A second PCR was applied to the resulting products in which dual indices (containing a 6-nt unique sequence to identify samples when pooled for sequencing) and sequencing adapters were incorporated using the Nextera XT Index kit (Illumina, United States), in order to generate complete libraries. The cycling conditions were 95°C for 5 min, followed by five cycles at 95°C for 1 min, 50°C for 1 min, 72°C for 1 min and a final 5 min extension at 72°C. Thereafter, AMPure XP beads were repeated to clean up the library. Finally, the resulting library in each sample was qualified and quantified using an Agilent 4200 TapeStation (Agilent, Santa Clara, CA, United States).

### Sequencing and Data Analysis

The 36 libraries were mixed in equimolar concentrations to generate a 4 nM library pool using 10 mM Tris (pH 8.5) as diluent. In addition, libraries were denatured with 0.2 N NaOH and diluted to a final concentration of 10 pM, including a 10% PhiX Control v3 (Illumina, Cat. No. FC-110-3001). DNA library was sequenced at Sequencing Unit in National Institute of Genomic Medicine (INMEGEN) by Illumina Miseq platform (Illumina, San Diego, CA, United States) as described by Caporaso et al. (2012). Illumina fastq reads were processed using the QIIME (quantitative insights into microbial ecology) software package (Caporaso et al., 2010). Forward and reverse reads were first merged using join_paired_ends.py script. The resulting sequences were filtered using split_libraries.py script with the following parameters: (*r* = 3, *p* = 0.75 total read length; *q* = 3; *n* = 0) as recommended by Bokulich et al. (2013). UCHIME algorithm was implemented to safely detect and remove chimeric sequences (Edgar et al., 2011). Briefly, sequences were clustered into operational taxonomic units (OTUs) using a 97% identity threshold with UCLUST tool, wrapped within QIIME (Edgar, 2010). Representative sequences were aligned against the Greengenes database (DeSantis et al., 2006), and taxonomy was assigned using the Ribosomal Database Project (RDP) classifier with a minimum support threshold of 80% (Wang et al., 2007). The taxonomic composition of the gut microbiota was assessed using METAGENassist (Arndt et al., 2012).

Community diversity was calculated by using the alpha_rarefaction.py script including estimator Chao1 (species richness), Shannon index (species diversity) and species metrics (Chao et al., 2006).

### Statistical Analysis

Statistical analyses of biochemical, anthropometrical, hormonal, and nutrients variables were performed using the IBM SPSS statistic version 22 (SPSS, Inc., Chicago, IL, United States) and R software^1^ (R Core Team, 2013). Parameters with normal distribution were compared by one-way ANOVA followed by Bonferroni *post hoc*. The non-parametric Kruskall–Wallis test and subsequent Tukey’s multiple comparison were used to determine which specific variables were significantly different among groups. Data were expressed as mean ± SD and median (25th–75th percentiles) and a *P*-value < 0.05 was considered statistically significant (**Supplementary Tables S1, S2**). Principal components analysis (PCA) was applied to biochemical, anthropometrical, hormonal, and nutrimental data. In order to standardize variables, a log-transformation using the prcomp function from the stats package was done (R Core Team, 2015).

Plots were generated using the ggplot2 package (Wickham, 2009). Parametric one-way ANOVA with Tukey test was used in order to detect significant differences on OTU and phylum, species richness or diversity indices. Correspondence analysis (CA) between bacterial communities (*Lachnospiraceae, Proteobacteria*, and *Prevotella*) and specific variables of diet intake (Energy and fat intake and beans consumption) was applied using FactoMineR (Lê and Husson, 2008) and factoextra (Kassambara and Mundt, 2016) packages. We used the partitioning around medoids (PAM) clustering algorithm and Jensen-Shannon divergence (JSD) to determine the genus-level relative abundance in the three groups and to cluster the gut microbial enterotypes.

The analysis among enterotypes and important diet parameters derived from the variables factor map (PCA), was carried out by a correlation matrix (Spearman’s rank correlation) constructed using the hmisc, and corrplot packages (Harrell and Dupont, 2016; Wei and Simko, 2016). The Lineal Discriminant Analysis (LDA) Effect Size (LEfSe) method was used to evaluate differences in microbial communities with an LDA score of at least 2 (Segata et al., 2011).

## Results

**FIGURE 1:**
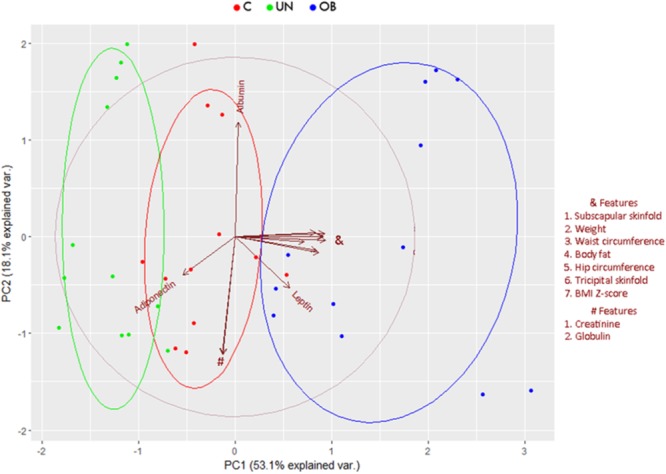
Principal component biplot of biochemical, anthropometric, and hormonal variables. The first principal component accounted for 53.1% while the second principal component accounted for 18.1% variance of the performance measures data. Arrows show the contribution and correlation of each variable on the PC1 and PC2. The central circle indicates the theoretical maximum extent of the arrows. The ellipses indicate 68% confidence intervals for the study groups.

Significant differences (*P* < 0.05) in the groups of undernourished, obese, and normal-weight Mexican school-age children in anthropometrical characteristics, such as BMI, waist circumference, tricipital and subscapular skinfolds, and body fat percentage. Regarding biochemical parameters, there were significant differences (*P* < 0.05) in glucose, triglycerides, insulin, ALT, and leptin levels (**Supplementary Table S1**). Division and discrimination achieved between the groups are shown in the Principal Component Analysis (**Figure 1**). The first principal component (PC1) accounted for 53.1% of the variance of the performance measures data, while the second principal component (PC2) accounted for 18.1%. The correlations between variables and the contribution of each variable to PC1 and PC2 are shown in **Supplementary Figure S1**.

### Inadequate Nutritional Intake Among the Obese and Undernutrition Groups

The obese group diet was characterized by high total fat intake, which comprised 36.1% of the total energy intake. The mean daily intake of saturated fatty acids (25.6 g), monounsaturated fatty acids (31.0 g), and polyunsaturated fatty acids (13.6 g) was notably higher than in the other groups (**Supplementary Table S2**). Carbohydrate intake accounted for about 66% of the total daily energy of undernourished children, due mainly to sugar consumption. However, the obese group consumed more kcal per kg per day than the other groups *kcal per kg per day*. **Supplementary Table S2** shows the daily intake of micronutrients (nine vitamins and six minerals). Obese children had higher sodium intake than the other two groups. We generated a variable factor map that shows the projection, contribution, and correlations between the dietary variables (**Supplementary Figures S2A,B**)

### Decrease in Alpha Diversity in Malnutrition Groups

**FIGURE 2:**
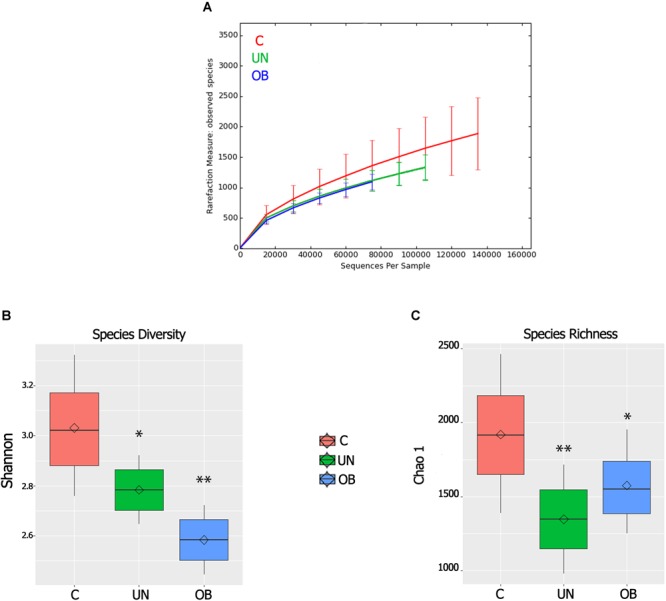
**(A)** Alpha rarefaction curves representing the observed number of species in the three study groups. The *y*-axis indicates the average number of OTUs per sample in each group. The error bars denote standard deviation. **(B)** Boxplots for comparison of species diversity (Shannon index) between the three study groups. **(C)** Boxplots for comparison of species richness (Chao1 index) between the three study groups. Diamonds indicate means and horizontal lines indicate medians ^∗^denotes *P* < 0.05 compared to the control group; ^∗∗^denotes *P* < 0.01 compared to the control group.

From the Illumina 250 bp paired-end sequencing of the amplicon targeting the V3–V4 region of 16S rRNA gene, we generated a total of 9,935,304 sequences, with a mean = 275,981 reads per sample. The three “non-phylogeny-based” metrics (the observed species, Chao1 and Shannon index) were used to describe alpha diversity (**Figures 2A–C**). The normal-weight group had more richness and observed species than the undernourished and obese groups (*P* = 0.007, *P* = 0.02, respectively). Regarding the Shannon diversity index, there was a significant difference between the normal-weight group and the undernourished (*P* = 0.02) and obese groups (*P* < 0.01), with the normal-weight group showing greater diversity.

### Characterization of Intestinal Microbiota

**FIGURE 3:**
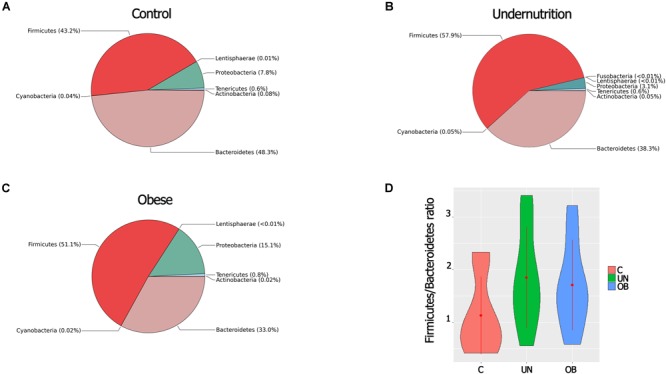
**(A)** Comparison of microbial composition at phylum-level among the three groups [Control **(A)**, Undernutrition **(B)**, Obese **(C)**]. The pie charts show the relative proportion of each phylum detected by METAGENassist analysis. *n* = 12 in each group. **(D)** Ratio F/B of the three study groups. Error bars indicate standard error of the mean.

A total of eight phyla were detected. The predominant phyla were *Firmicutes* and *Bacteroidetes*, followed by *Proteobacteria, Actinobacteria, Tenericutes, Actinobacteria*, and *Lentisphaerae*, which were less abundant. In regard to phyla, *Firmicutes* showed greater abundance in the undernourished group (57.9%) than in the normal-weight group (43.2%) (*P* = 0.028). The intestinal microbiota of children in the undernourished and obese groups had lower proportions of *Bacteroidetes* (38.3% and 33%, respectively) compared to the normal-weight group (48.3%) (**Figures 3A–C**).

The ratio of *Firmicutes* to *Bacteroidetes* (F/B) was greater in the malnourished group than in the normal-weight group. However, these differences were not significant (**Figure 3D**). *Proteobacteria* were substantially more abundant in the obese group (15.1%) than in the undernourished group (3.1%) (*P* = 0.002). The most abundant genera belonged to the three dominant phyla: three genera belonged to *Bacteroidetes, Ruminococcus*, and *Roseburia* belonged to *Firmicutes*, and *Sutterella* and *Succinivibrio* belonged to *Proteobacteria* (**Supplementary Figure S3**).

**FIGURE 4:**
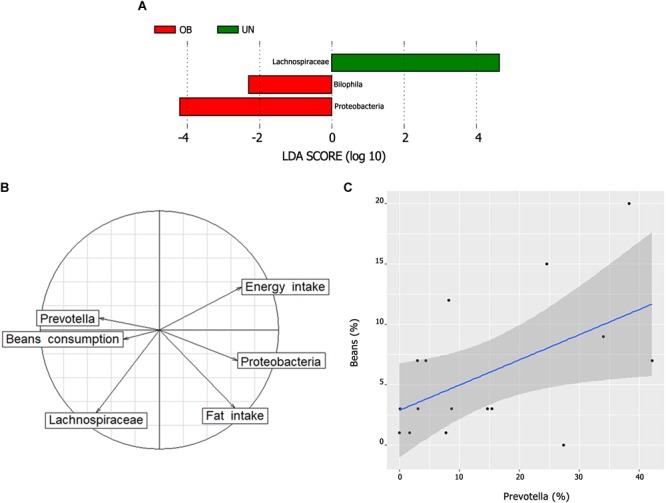
**(A)** Bacterial taxa differentially represented between the undernutrition and obesity groups identified by linear discriminant analysis (LDA) effect size (*LEfSe*). Only taxa with an alpha value of 0.05 and with an LDA score of at least 2 are shown. **(B)** Biplot of correspondence analysis (between important bacterial lineages and relevant dietary parameters). Arrows pointing to the same position indicate a positive correlation and arrows pointing to an opposite position indicate a negative correlation. **(C)** Correlation between beans consumption and *Prevotella*. The graph shows a positive correlation between the two variables (ρ = 0.52, *P* = 0.03) including a regression line and a 95% confidence interval represented by the shaded area.

We used the LEfSe algorithm to determine whether any taxa at different taxonomic levels were enriched in the undernutrition and obese groups (**Figure 4A**). *Proteobacteria* and *Bilophila* were overrepresented in the obese group (LDA score ≥ 4 and LDA score ≥ 2, respectively), while *Lachnospiraceae* was enriched in the undernourished group (LDA score ≥ 4.5). *Proteobacteria* correlated positively with total fat intake (ρ = 0.48, *P* = 0.01), whereas *Lachnospiraceae* correlated negatively with energy intake (ρ = -0.77, *P* = 0.02) (**Figure 4B**) and correlated positively with leptin (ρ = 0.24, *P* = 0.001) (**Supplementary Figure S5**). There was also a positive correlation between *Prevotella* and bean consumption (ρ = 0.52, *P* = 0.03) (**Figures 4B,C**).

### Association of Enterotypes 1 and 2 With Dietary Intake

*Bacteroides* (enterotype 1) showed a higher relative abundance in the control group compared to the undernourished and obese groups, while the relative abundance of *Prevotella* (enterotype 2) was higher in the undernourished group than in the control and obese groups (**Supplementary Figure S3**).

**FIGURE 5:**
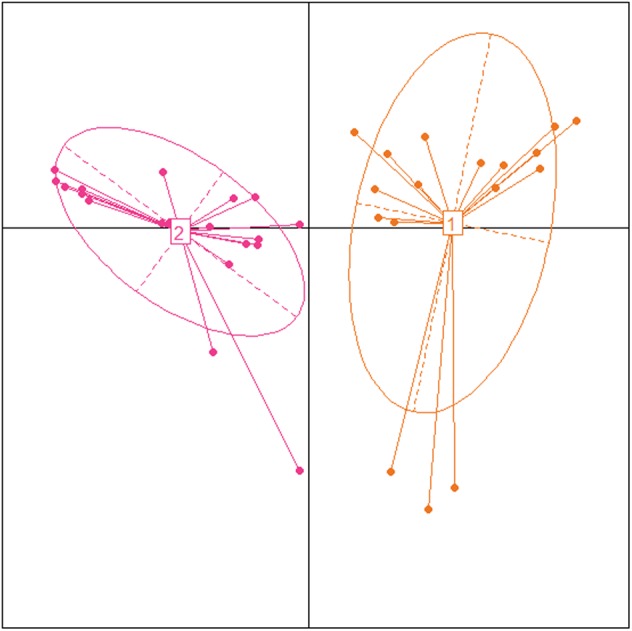
Identification of enterotypes in Mexican children using Principal Coordinate Analysis. Samples colored by enterotype: orange color corresponds to enterotype 1 (*Bacteroides*) and pink color corresponds to enterotype 2 (*Prevotella*).

**FIGURE 6:**
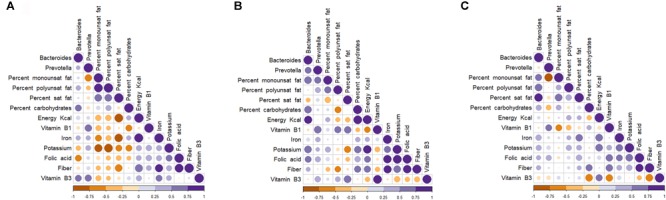
Spearman (rank) correlation matrix between enterotypes and dietary intakes in fecal samples of selected groups of children. Control **(A)**, Undernutrition **(B)**, Obese **(C)**. Strong correlations are indicated by big circles, whereas weak correlations are indicated by small circles. Colors in scale bar denote the type of correlation: 1 indicates perfect positive correlation (dark purple) and –1 indicates perfect negative correlation (dark yellow).

A total of 19 (52.7%) of the 36 samples were assigned to enterotype 1, and 17 (47.2%) were assigned to enterotype 2 (**Figure 5**). These enterotypes did not show significant correlations with any of the biochemical, hormonal, or anthropometric parameters (**Supplementary Figure S4**). We also investigated the correlations between enterotypes 1 and 2 and the dietary patterns of each group. *Bacteroides* correlated positively with fat intake and with carbohydrate consumption (**Figure 6**).

## Discussion

Previous studies have shown that obesity is associated with changes in gut microbiota (Ridaura et al., 2013), but few studies have investigated the microbiota composition in undernourished children, including those affected by poverty in developing countries. The undernourished population tends to shift from undernutrition to overnutrition, but the mechanisms underlying the associated alterations remain unknown (Barquera et al., 2007). However, they may involve dysbiosis in the gut microbiota.

The present study categorized children into normal-weight, obese, and undernourished groups. Independent of nutrition status, the gut microbiota of the children were dominated by *Bacteroidetes* (including *Bacteroides* and *Prevotella* genera) and *Firmicutes* (including *Clostridium, Enterococcus, Lactobacillus*, and *Ruminococcus*), which account for more than 90% of the phylogenetic lineages (Sagheddu et al., 2016).

Microbiota diversity and richness was lower in the undernourished and obese groups than in the normal-weight group. Interestingly, this pattern has also been reported in undernourished mice (Preidis et al., 2015). Studies indicate that individuals with low intestinal bacterial richness have more overall adiposity and insulin resistance and gain more weight over time (Le Chatelier et al., 2013). Indeed, significant decreases in diversity and phylum-level changes have been associated with obesity (Turnbaugh et al., 2009a). Ley et al. (2006) found a higher F/B ratio in obese people than in thin people. In the present study, the F/B ratio was higher in the undernutrition group than in the other groups; however, this difference was not significant.

*Bacteroidetes* have a very large repertoire of genes that are involved in the acquisition and metabolism of polysaccharides (Mahowald et al., 2009). *Bacteroidetes* can easily adapt to any environmental niche, owing in part to the plasticity of their genomes, which undergo continuous genetic rearrangements, duplications, and lateral gene transfers between species (Thomas et al., 2011). The relative abundance of *Bacteroidetes* was lowest in the obese group and was higher in the normal-weight group compared to the undernourished group. This is in accordance with data from undernourished neonatal mice, who have a relatively lower proportion of *Bacteroidetes* compared to controls and are deficient in multiple microbial pathways, including the *N*-glycan pathway. This deficiency may result in less efficient energy extraction from non-digestible polysaccharides or diet fiber (Preidis et al., 2015).

In addition, we found that the F/B ratio in the gut microbiota of the undernourished group was higher than in the obese and normal-weight groups. This might be because the undernourished group had a diet high in sugar and low in fiber, which could predispose them to future obesity. There is evidence that high-glucose or -sucrose consumption induces changes in gut microbiota, increasing the F/B ratio (Magnusson et al., 2015; Do et al., 2018). On the other hand, a so-called Western diet, which is high in sugar and fat, has been associated with an overgrowth of *Firmicutes* and a parallel decrease in *Bacteroidetes* (Turnbaugh et al., 2009b). The Western lifestyle, including the diet, is associated with high incidences of chronic diseases, such as cardiovascular disease and type II diabetes, which individually and collectively have a hefty socioeconomic burden (Turnbaugh et al., 2009b). Most Western populations have omnivorous diets that are rich in refined food and of poor nutritional quality (Conlon and Bird, 2015).

We found that the *Lachnospiraceae* family within the phylum *Firmicutes* was significantly overrepresented in undernourished children and correlated negatively with energy intake. Early blooms of this family have been associated with adiposity, weight gain, and diabetes (Cho et al., 2012; Kameyama and Itoh, 2014). According to Turnbaugh et al. (2006), the gut microbiomes of obese people with the lowest levels of microbiota diversity and richness had higher energy harvesting. The mechanisms involved in this phenomenon are not yet clear, but a recent study showed that *Lachnospiraceae*, which was detected in the microbiomes of animals with more efficient energy harvesting, could be a contributing factor (Shabat et al., 2016). Considering these data, we hypothesize that having high *Lachnospiraceae* levels contributes to a process of adaptation that protects against poor nutrition. In this context, although the microbial alpha diversity was low in undernourished children, this type of bacteria helps improve the energy balance, suggesting that the *Lachnospiraceae* family might serve as a metabolic regulator in individuals with an undernourished phenotype. As mentioned above, we found that *Firmicutes*, particularly *Lachnospiraceae*, was enriched in the undernourished group. Notably, the presence of *Lachnospiraceae* is closely related to obesity. Hence, we hypothesize that an expansion of *Firmicutes* could improve energy homeostasis and protect against undernutrition, but it could also have negative cumulative effects on the children’s health in the future. Interestingly, *Lachnospiraceae* abundance correlated with leptin levels; moreover, malnourished children had higher concentrations of leptin than normal-weight children. Studies indicate that independent of body fat, high serum leptin levels may be an indicator of increased leptin resistance, which predisposes children who are at high risk of adult obesity to weigh more and to have more body fat during childhood (Fleisch et al., 2007). According to the nutrition transition theory, populations tend to shift from undernutrition to overnutrition as they experience the dietary and demographic changes associated with socioeconomic development (Barquera et al., 2007). Undernutrition in early life results in an increased risk of hyperinsulinemia, high blood pressure, obesity, diabetes, and cardiovascular diseases in adulthood (Guerrant et al., 2013). In Mexico, child stunting is one of the biggest nutritional public health problems, particularly in underserved groups, including those in rural areas (Kroker-Lobos et al., 2014). Interestingly the prevalence of stunting in Mexico has decreased in recent decades, but this has been coupled to a dramatic change in the prevalence of overweight and obesity in children (Shamah-Levy et al., 2017). Thus, our data suggest that *Lachnospiraceae* may be linked to increased energy harvest by gut microbiota. Additional studies are needed to determine whether the abundance of *Lachnospiraceae* early in life predisposes undernourished children to obesity later in life.

Our results showed that the obese group had higher levels of *Proteobacteria*, and we also found a positive correlation between *Proteobacteria* and fat intake. Preliminary investigation showed that an increase in *Proteobacteria* represents a risk factor for human health, including dysbiosis, and therefore the abnormal growth of *Proteobacteria* may represent an imbalance in the gut microbial community and be a potential marker of disease risk (Shin et al., 2015). Moreover, the combination of low alpha diversity and *Proteobacteria* expansion may reflect intestinal dysbiosis in obese children. A recent study in mice found an association between increased abundance of *Bilophila* and fat feeding, inflammation, and colitis (Devkota et al., 2012). In the present study, the *Bilophila* level was increased in the obese group of children.

To determine whether the enterotypes of the human gut microbiome are linked to the Western diet, we analyzed possible correlations between *Bacteroides* and *Prevotella* and energy and macronutrient intake. The *Bacteroides* enterotype was positively associated with energy intake and fat intake, including the intake of monounsaturated, polyunsaturated, and saturated fat. *Bacteroides* are associated with consumption of a long-term fat-enriched diet (Lim et al., 2014). These results indicate that, despite the alterations of the gut microbiota in undernourished and obese children, enterotype 1 (*Bacteroides*) can be considered a potential bacterial marker of a Western diet that predisposes them to chronic diseases. A recent study showed that *Prevotella* was associated with long-term consumption of dietary fiber. Likewise, we found a strong, positive correlation between *Prevotella* and bean consumption. Beans are one of the most widely consumed foods in the Mexican population (Mojica and de Mejia, 2015).

This is the first study to examine the gut microbial community structure in undernourished and obese Mexican children living in low-income neighborhoods. Our analysis revealed distinct taxonomic profiles for undernourished and obese children.

## Author Contributions

EM-S, MG-S, BP-G, and MM conceived and designed the experiments, analyzed the data, and wrote the paper. EM-S, MO-L, MG-S, and BP-G performed the experiments.

## Conflict of Interest Statement

The authors declare that the research was conducted in the absence of any commercial or financial relationships that could be construed as a potential conflict of interest.

## Footnotes

1 https://www.r-project.org/

## Supplementary Material

FIGURE S1 Scree plot graphs derived from principal components analysis. Purple bars show the first selected variables that explain 95% of the total variance. The *x*-axis contains the principal components. The *y*-axis contains the fraction of the explained total variance, indicated by bold horizontal red lines that define the most important parameters and relegates the variables without relevance in the PCA.

FIGURE S2 PCA variables factor map representing projection of variables on the plane defined by the first two dimensions based on 28 variables. **(B)** Variable contribution for Dimensions 1 and 2 with the most variance percentage (57%).

FIGURE S3 Comparison of taxonomic composition at genus-level among the three groups. The pie charts show the overall microbiota structure for each group at the genus level. **(A)** Control; **(B)** Under nutrition and **(C)** Obese. *n* = 12 in each group.

FIGURE S4 Spearman (rank) correlation matrix between enterotypes and anthropometrical, hormonal, and biochemical variables which derived from PCA (**Figure 1**). Strong correlations are indicated by big circles whereas weak correlations are indicated by small circles.

FIGURE S5 Correlation between serum leptin concentration and Lachnospiraceae. The graph shows a positive correlation between the two variables (ρ = 0.2403, *P* = 0.001) including a regression line and a 95% confidence interval represented by the shaded area.

TABLE S1 Anthropometric, biochemical, and hormonal characteristics in malnutrition and normal-weight groups. Data are mean ± SD and median (25th–75th percentiles). ^a^Statistically significant difference compared with the normal-weight group at *p* < 0.05. ^b^Statistically significant difference compared with the undernutrition group at *p* < 0.05. ^∗^Raw data; ^†^z (standard) score vs. normal-weight, (*p* < 0.001).

TABLE S2 Daily energy and dietary assessment in malnutrition and normal-weight groups. Data are mean ± SD and median (25th – 75th percentile). ^a^Statistically significant difference compared with the normal-weight group at *p* < 0.05. ^b^Statistically significant difference compared with the undernutrition group at *p* < 0.05.
