# Corrigendum: Altered Gut Microbiota and Compositional Changes in *Firmicutes* and *Proteobacteria* in Mexican Undernourished and Obese Children

**DOI:** 10.3389/fmicb.2018.02693

**Published:** 2018-11-16

**Authors:** Eder Orlando Méndez-Salazar, María Guadalupe Ortiz-López, María de los Ángeles Granados-Silvestre, Berenice Palacios-González, Marta Menjivar

**Affiliations:** ^1^Unidad de Genómica de Poblaciones Aplicada a la Salud, Facultad de Química UNAM-INMEGEN, Instituto Nacional de Medicina Genómica, México City, Mexico; ^2^Laboratorio de Endocrinología Molecular, Hospital Juárez de México, México City, Mexico; ^3^Facultad de Química, Universidad Nacional Autónoma de México, México City, Mexico; ^4^Unidad de Vinculación Científica de la Facultad de Medicina UNAM-INMEGEN, Instituto Nacional de Medicina Genómica, México City, Mexico

**Keywords:** Mexican children, microbiota, *Firmicutes*, undernourished, obesity

In the original article, there was an error. The percentages of malnutrition and obesity are incorrect. A correction has been made to the sections below.

## Abstract

Mexico is experiencing an epidemiological and nutritional transition period, and Mexican children are often affected by the double burden of malnutrition, which includes undernutrition (13.6% of children) and obesity (15.3%). The gut microbiome is a complex and metabolically active community of organisms that influences the host phenotype. Although previous studies have shown alterations in the gut microbiota in undernourished children, the affected bacterial communities remain unknown. The present study investigated and compared the bacterial richness and diversity of the fecal microbiota in groups of undernourished (*n* = 12), obese (*n* = 12), and normalweight (control) (*n* = 12) Mexican school-age children. We used next-generation sequencing to analyze the V3-V4 region of the bacterial 16S rRNA gene, and we also investigated whether there were correlations between diet and relevant bacteria. The undernourished and obese groups showed lower bacterial richness and diversity than the normal-weight group. Enterotype 1 correlated positively with dietary fat intake in the obese group and with carbohydrate intake in the undernourished group. The results showed that undernourished children had significantly higher levels of bacteria in the *Firmicutes* phylum and in the *Lachnospiraceae* family than obese children, while the *Proteobacteria* phylum was overrepresented in the obese group. The level of *Lachnospiraceae* correlated negatively with energy consumption and positively with leptin level. This is the first study to examine the gut microbial community structure in undernourished and obese Mexican children living in low-income neighborhoods. Our analysis revealed distinct taxonomic profiles for undernourished and obese children.

## Introduction, paragraph number: 2

Mexico is going through a nutritional transition that affects school-age children, who bear the double burden of malnutrition, which includes undernutrition and obesity (13.6% and 15.3%, respectively) (Shamah-Levy et al., 2017). An undernourished population often moves from undernutrition to overnutrition, resulting in weight gain and central adiposity (Barquera et al., 2007). There is a risk of obesity in populations who move from famine early in life to abundance or even to excessive nutrition in adulthood (Martorell et al., 2001; Tzioumis and Adair, 2014). The mechanisms underlying these alterations remain unknown. However, low-income families may have an obesogenic environment due to the enrichment of sugar and edible oils in inexpensive food, resulting in a diet that is energy dense but micronutrient-poor that could alter their gut microbiome (Sawaya et al., 2003; David et al., 2014; Doak et al., 2016).

The authors apologize for this error and state that this does not change the scientific conclusions of the article in any way. The original article has been updated.

## Conflict of interest statement

The authors declare that the research was conducted in the absence of any commercial or financial relationships that could be construed as a potential conflict of interest.
