# Supplementary figures and images for: Altered Gut Microbiota and Compositional Changes in Firmicutes and Proteobacteria in Mexican Undernourished and Obese Children
Source: Front Microbiol. 2018 Oct 16;9:2494. doi: 10.3389/fmicb.2018.02494 (PMC6198253; doi:10.3389/fmicb.2018.02494)

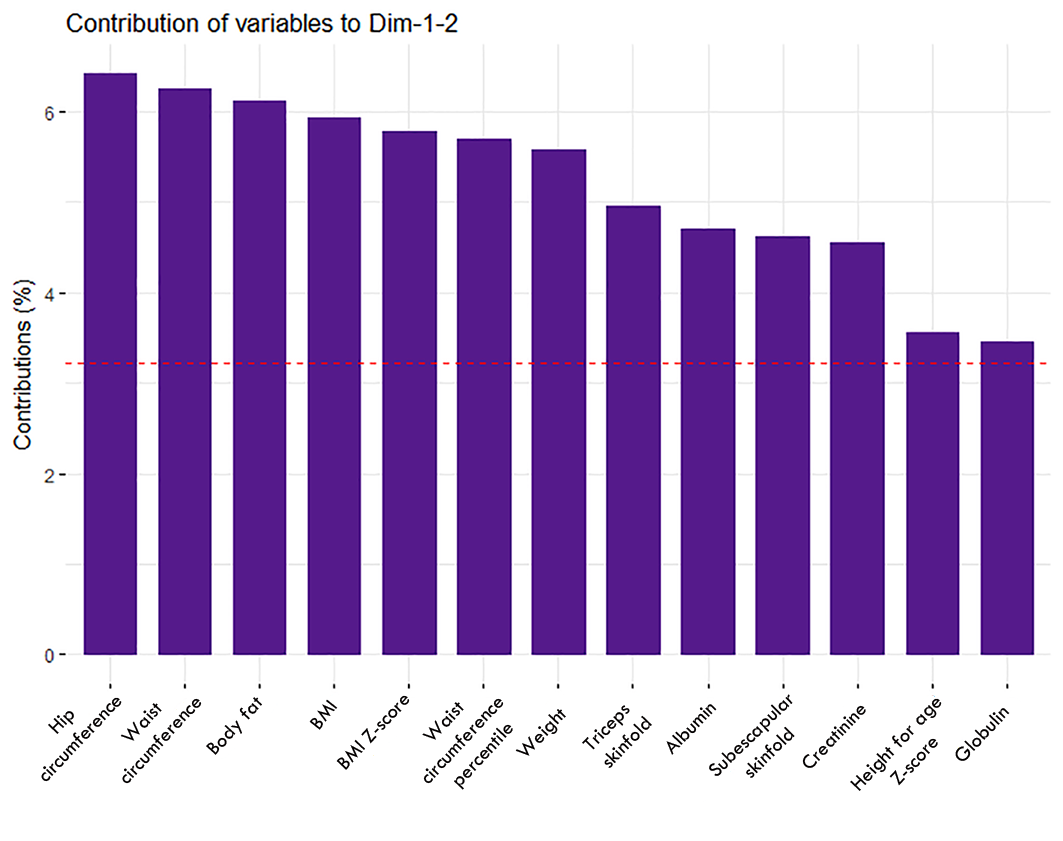

Supplement: FIGURE S1 — Scree plot graphs derived from principal components analysis. Purple bars show the first selected variables that explain 95% of the total variance. The x-axis contains the principal components. The y-axis contains the fraction of the explained total variance, indicated by bold horizontal red lines that define the most important parameters and relegates the variables without relevance in the PCA. [file Image_1.TIF]

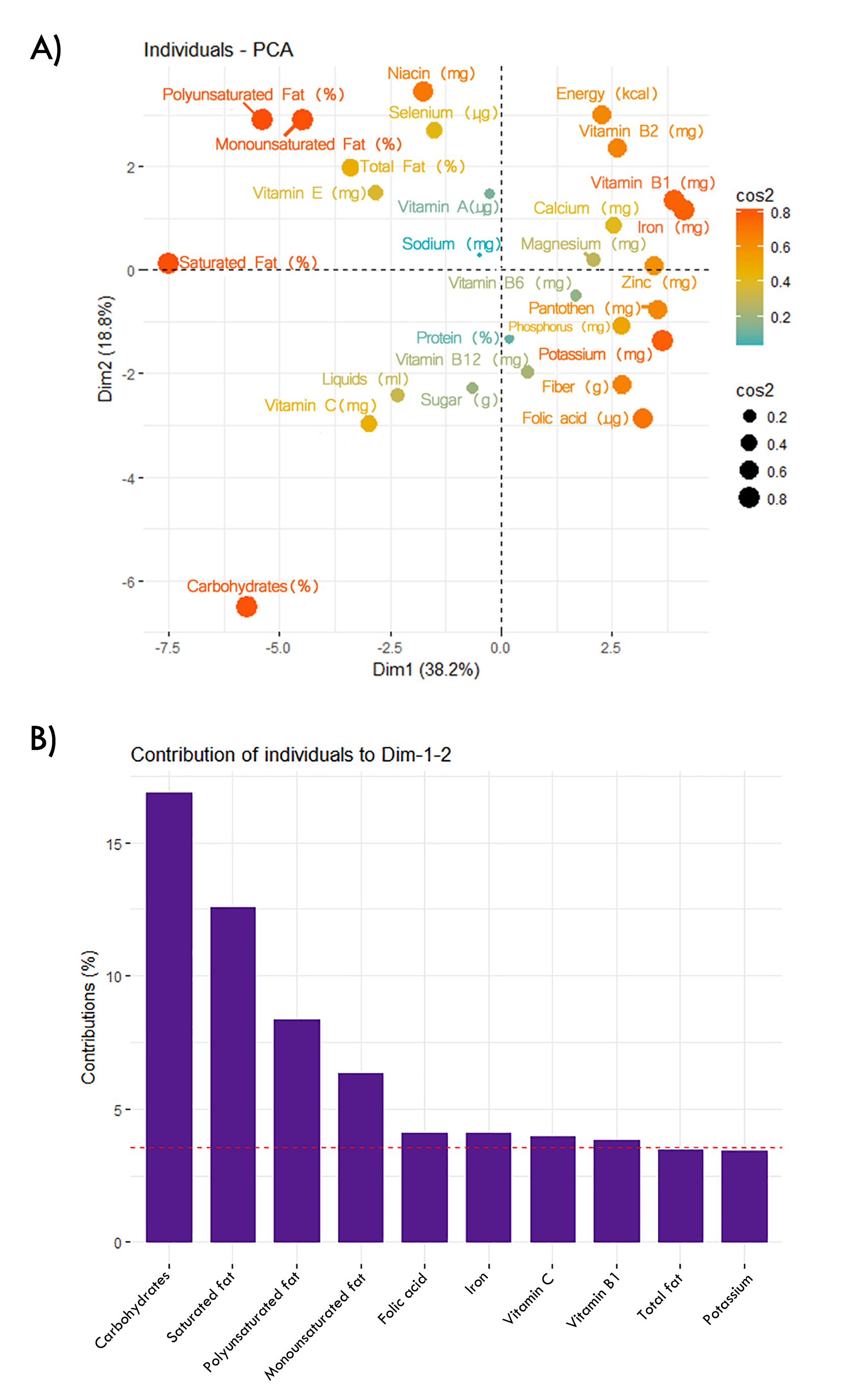

Supplement: FIGURE S2 — PCA variables factor map representing projection of variables on the plane defined by the first two dimensions based on 28 variables. (B) Variable contribution for Dimensions 1 and 2 with the most variance percentage (57%). [file Image_2.TIF]

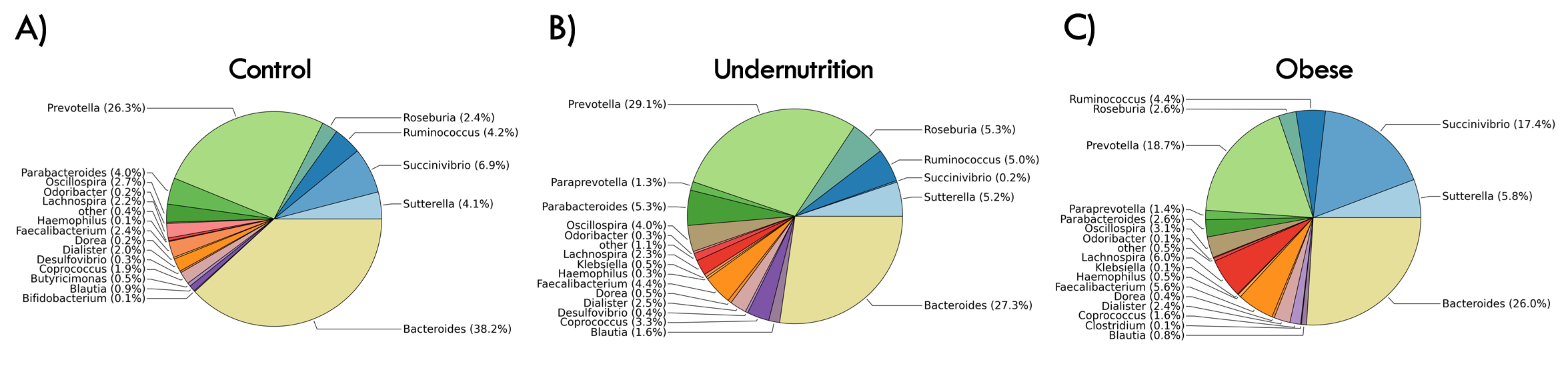

Supplement: FIGURE S3 — Comparison of taxonomic composition at genus-level among the three groups. The pie charts show the overall microbiota structure for each group at the genus level. (A) Control; (B) Under nutrition and (C) Obese. n = 12 in each group. [file Image_3.TIF]

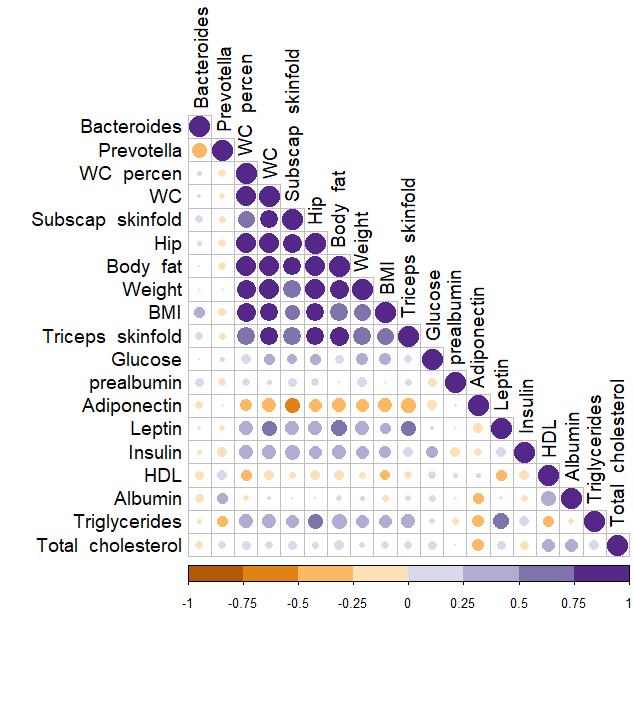

Supplement: FIGURE S4 — Spearman (rank) correlation matrix between enterotypes and anthropometrical, hormonal, and biochemical variables which derived from PCA (Figure 1). Strong correlations are indicated by big circles whereas weak correlations are indicated by small circles. [file Image_4.TIF]

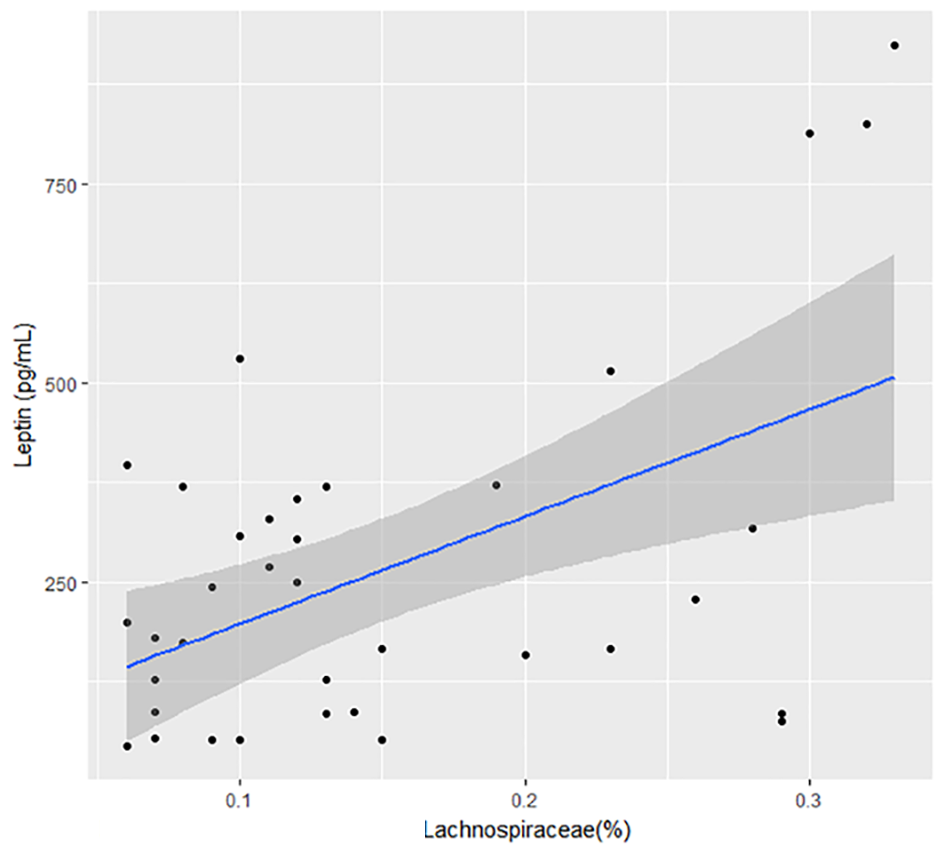

Supplement: FIGURE S5 — Correlation between serum leptin concentration and Lachnospiraceae. The graph shows a positive correlation between the two variables (ρ = 0.2403, P = 0.001) including a regression line and a 95% confidence interval represented by the shaded area. [file Image_5.TIF]
