# Supplementary material for: Altered Gut Microbiota and Compositional Changes in Firmicutes and Proteobacteria in Mexican Undernourished and Obese Children
Source: Front Microbiol. 2018 Oct 16;9:2494. doi: 10.3389/fmicb.2018.02494 (PMC6198253; doi:10.3389/fmicb.2018.02494)
Supplement: TABLE S1 — Anthropometric, biochemical, and hormonal characteristics in malnutrition and normal-weight groups. Data are mean ± SD and median (25th–75th percentiles). aStatistically significant difference compared with the normal-weight group at p < 0.05. bStatistically significant difference compared with the undernutrition group at p < 0.05. ∗Raw data; †z (standard) score vs. normal-weight, (p < 0.001). [file Data_Sheet_1.PDF]

Table S1

	Control	Undernutrition	Obese
N	12	12	12
Age (years)	10.2±0.4	10.0 ±1.2	9.6±0.8
Sex (male/female)	8/4	4/8	8/4
Height cm	140.1(134.3-147.7)	129.9 (123.1-133.5)[a]	132.5 (127.6-139.3)[b]
Weight (kg)	32.8(30.7-38.3)	28.1 (23.8-32.9)[a]	39.9 (31.2-53.5)[a,b]
BMI (Kg/m^2)	17.1±0.6	15.5±1.3	28.6 ±3.1[a,b]
BMI z-score †	0.02± 1.3	-0.68 ±0.7[a]	2.4±0.3[a,b]
Height-for-age, Z †	0.01±1.1	-2.4±0.3 [a]	-0.2± 0.8[b]
Waist circumference (cm)	60.7(58.0-64.5)	54.0 (53.5-56.5)[a]	72.5 (67.0-82.0)[a,b]
Waist circumference (percentile)	30.3(25-50)	14.2 (10-25)[a]	75 (75-90) [a,b]
Triceps skinfold (mm)	12.±3.4	9.0±5.0	17.6 ±5.6[a,b]
Subscapular skinfold (mm)	11.5(8.2-15.7)	10.0 (6.0-14.0)	17.5 (14.5-22.7)[a,b]
Body fat % (male/female)<br>(triceps + subscapular) skinfold sum	23/24	18/20	35/33
Glucose (mg/dL)	88.5(82.5-92.5)	79.0 (76.5-85.0)[a]	86.5(83.7-89.7)[b]
Total cholesterol (mg/dL)	134.3(124.5-144.7)	121.8(109.5-131.0)	135.5(124.5-144.7)
Triglycerides (mg/dL)	53.0 (46.0-70.0)	67(65.0-81.0)[a]	91.0 (64.0-112.5)[a,b]
HDL-cholesterol (mg/dL)	53.4±6.7	42.3±9.1	44.0±6.8
LDL-cholesterol (mg/dL)	91.4±22.7	85.2±7.9	126.0±15.7
VLDL- cholesterol (mg/dL)	12.0 (9.5-14.7)	13.0 (13.0-16.5)	17.0 (13.7-20.7)[a]
BUN (mg/dL)	9.3±3.2	10.2±3.0	11.1±2.7
Urea (mg/dL)	20.0±6.7	21.8±6.5	24.0±5.8
Creatinine (mg/dL)	0.7±.06	0.8±.07	0.7±.05
Uric Acid (mg/dL)	3.9(3.1-4.7)	4.9(4.1-5.2)	4.8 (4.1-5.6)[a]
Total Proteíns (g/dL)	7.3±.34	7.7±.34	7.8±.38
Albumin (mg/dL)	3.9±0.07	4.0±.13	4.1±.09
Globulin (mg/dL)	3.4±.29	3.7±.29	3.6±.34
AST (U/L)	30.6±3.7	30.8±2.5	34.6±8.8
ALT (U/L)	17(11.7-19.5)	14(11.0-19.5)	25 (16.0-38.2)[a,b]
Insulin (*uUI/mL*)	4.7(3.1-6.6)	4.4(2.2-5.9)	5.0 (4.3-18.2)[b]
HOMA-IR	1.0(0.6-1.4)	0.7(0.5-0.9)	1.1(1.0-3.1)[b]
Leptin (pg/mL)	106.2 (52.2-294.1)	212.7(144.4-326.0)[a]	571.2 (287.8.-850.5)[a,b]
Adiponectin (ng/mL)	10.9±2.3	13.0±4.1	7.3±2.7
Prealbumin mg/mL	23.2±3.5	21.2±2.3	22.6±3.1
Transferrin µg/dL	265.8±22.8	245.3±38.8	289.5±40.2
Hemoglobin (g/dL)	13.9±.65	14.4±1.4	14.8±.53
VCM (fL)	82.5±1.3	80.9±4.5	86.4±3.9
