# Supplementary material for: Altered Gut Microbiota and Compositional Changes in Firmicutes and Proteobacteria in Mexican Undernourished and Obese Children
Source: Front Microbiol. 2018 Oct 16;9:2494. doi: 10.3389/fmicb.2018.02494 (PMC6198253; doi:10.3389/fmicb.2018.02494)
Supplement: TABLE S2 — Daily energy and dietary assessment in malnutrition and normal-weight groups. Data are mean ± SD and median (25th – 75th percentile). aStatistically significant difference compared with the normal-weight group at p < 0.05. bStatistically significant difference compared with the undernutrition group at p < 0.05. [file Data_Sheet_2.PDF]

Table S2

	Control (12)	Undernutrition (12)	Obese (12)
Energy (Kcal/day)	1812(1353.7-2386.5)	1398(922.0-1613.0)	2566 (1644.5-2939.5)[b]
Energy (Kcal/kg/day)	55.2 (44.0-62.3)	49.7(38.7-59.0)	64.3 (52.7-72.9)[b]
Carbohydrates (gr/day)	231.6±96.9	209.0 ±57.0	280.3 ±107.3
Carbohydrates (% of energy)	48.5±10.3	61.1±5.4[a]	47.7 ±10.6[b]
Total sugar (gr/day)	35.7(9.5-57.2)	46.1(15.2-49.6)	42.5(22.9-76.3)
Protein (gr/day)	69.1±25.3	53.3±18.5	96.5±41.6[b]
Protein (% of energy)	15.1±3.8	17.2±3.7	16.6±4.1[b]
Total fat (gr/day)	72.0.1±21.4	33.0±15.3[a]	94.2±40.6[b]
Fat (% of energy)	36.1±8.1	21.4±3.3[a]	35.8±6.8[b]
Saturated fat (g/day)	19.8±4.9	12.7±6.1	25.6±10.8[b]
Saturated fat (% of energy)	11.0±5.4	8.4±2.7	10.2±2.8
Monounsaturated fat (g/day)	18.0±8.7	9.5±7.8	31.0±12.7[b]
Monounsaturated fat (% of energy)	9.5±4.0	6.4±3.9	11.5±5.1
Polyunsaturated fat (g/day)	6.2±3.4	4.0±2.3	13.6 ±1.1[b]
Polyunsaturated fat (% of energy)	3.5±2.2	2.7±1.6	5.0±3.0
Total dietary fiber (g/day)	14.2±2.3	11.1±3.5	17.8±3.1
Líquids (mL/day)	1425.5(988.0-1862.9)	1077.2(780.8-1373.7)	1149.2(923.6-1374.8)
**Vitamins**			
Vitamin A (μg)	367(257-407.7)	398(311-503)	1543 (678-1872.2)[a,b]
Thiamin (mg)	0.9±0.4	1.0±0.5	1.4±0.6
Rivoflavin (mg)	1.2±0.3	1.0±0.6	1.6±0.5
Niacin (mg)	10.3(8.5-12,7)	9.7(7.6-11.1)	14.6(7.5-17.6)
Vitamin B-6 (mg)	0.6±0.4	0.8±0.3	1.1±0.6
Folic acid (μg)	130(57.2-164.4)	85(30.6-145.5)	150(45.8-292.2)
Vitamin B-12 (μg)	2.0±0.6	2.3±1.1	2.1±0.7
Vitamin B5 (mg)	2.4±0.9	2.4±2.1	2.5±0.6
Vitamin C (mg)	19.6(4.4-58.2)	30.2(11.0-75.4)	52.8(16.0-98.3)
**Minerals**			
Calcium (mg)	852.5±348.1	725.4±351.7	914.1±390.4
Iron (mg)	10.0±5.0	10.4±4.8	14.8±7.1
Magnesium (mg)	197.5(160.7-418.0)	227(121.0-648.0)	277(178.7-587.7)
Phosphorus (mg)	745(537.7-899.5)	799(131.0-1604.0)	924(578.2-946.7)
Sodium (mg)	1839±781.5	1138.4±514.1	2211.2±970.5[a,b]
Zinc (mg)	4.4±1.8	5.7±3.2	6.0±2.7
